# Supplementary material for: Pixel-level Bayer-type colour router based on metasurfaces
Source: Nat Commun. 2022 Jun 7;13:3288. doi: 10.1038/s41467-022-31019-7 (PMC9174490; doi:10.1038/s41467-022-31019-7)
Supplement: Supplementary file 1 — Supplementary information [file 41467_2022_31019_MOESM1_ESM.pdf]

Supplementary information for

Pixel-level Bayer-type color router based on metasurfaces

Xiujuan Zou[1,*], Youming Zhang[2,†,*], Ruoyu Lin[1], Guangxing Gong[1], Shuming Wang[1,3,4,†], Shining Zhu[1,3,4], and Zhenlin Wang[1,4,†]

[1]National Laboratory of Solid State Microstructures, School of Physics, Nanjing University, Nanjing, 210093, China

[2]Huawei Technologies Co., Ltd., Shenzhen, 518129, China

[3]Key Laboratory of Intelligent Optical Sensing and Manipulation, Ministry of Education, Nanjing University, Nanjing, 210093, China

[4]Collaborative Innovation Center of Advanced Microstructures, Nanjing, 210093, China

*These authors contributed equally: Xiujuan Zou, Youming Zhang.

†Corresponding author. Emails: zhangyouming3@huawei.com, wangshuming@nju.edu.cn, zlwang@nju.edu.cn.

This PDF file includes:

Materials and Methods

Supplementary Text

Supplementary Figures 1 to 22

Supplementary Note 1. Numerical simulation analysis

The final optimized structure is shown in Supplementary Figure 2. Using the optimized metasurface, we simulated the transmittance of incident light on the four quadrants of the unit cell after passing through the pixel-level MBCR (Fig. 2f-h) and the power flow density distribution maps of the transmitted light on the focal plane for the vertical polarization in Supplementary Figure 3a at 660 nm, 540 nm and 450 nm, respectively. Similarly, power flow density distributions on the focal plane for the horizontal polarization in Supplementary Figure 3b are the same as the diagonal inversion of the field distribution in the vertical polarization case, because of the symmetry of the metasurface. In Supplementary Figure 3 or Fig. 2g, the power flow density distribution for green light looks not well collected, because the calculation window selected is the whole green pixel area when calculating the fitness function. Furthermore, we also plot the power flow $|P|$ maps in the z direction at λ =660 nm, 540 nm, and 450 nm shown in Supplementary Figure 4, which is consistent with the top view of Poynting vector maps in the manuscript of Fig. 2c-e and light is effectively routed to the corresponding pixels.

In order to understand the physical mechanism of light routing for the optimized pattern, optical mode analysis is conducted at the three wavelengths (λ =450 nm, 540 nm, and 660 nm), as shown in Supplementary Figure 5a-5c. It shows that the top-view electric field $|E|$ distributions are enhanced inside the nanostructures of the metasurface for shorter wavelengths, while for longer wavelengths are enhanced in the gaps between nanostructures.

Supplementary Note 2. Figure of merit of the optimization and computing resources

Some intermediate results during GA algorithms are provided as follows. These initial solutions are randomly generated with a filling factor of 0.1. The number of solutions for each generation is set to 10. The figure of merit (FOM) line is shown in Supplementary Figure 10. The vertical axis in Supplementary Figure 10 is the generation number of iterations. The solid red line shows how the global best FOM evolves with iteration. The dashed grey line shows how the best FOM of each generation evolves with iteration. Parts of the specific structures for the best global FOMs are also shown in the insets, from which we can see how the structure evolves as the FOM increases.

Some hyperparameters as well as the computer resources of GA algorithms are provided.

Specifications of the workstations used for the numerical optimization and some other details of the optimization processes are set as follows:

a) CPU: Intel Xeon Gold 6134 CPU @ 3.20 GHz
b) RAM: 128 GB
c) Mesh size: 20 nm (late stage) & 50 nm (early stage)
d) Simulation time: 20 nm mesh: about 5 minutes 45 seconds per simulation; 50 nm mesh: about 55 seconds per simulation
e) Boundary conditions: X & Y directions: Periodic; Z direction: Perfect matching layer (PML)

Supplementary Note 3. MBCR characterization and efficiency calculation

In the prepared sample, it is found that the period of the fabricated structures can be strictly controlled and the central positions of every nano-pillars remain unchanged, but the side lengths of the nano-pillars are enlarged slightly and the maximum magnification is ~10% shown in Supplementary Figure 12a. We simulated the spectral efficiencies when the size expansion of the nano-pillars was 12% - 20% (a=140 nm ~150 nm) and other parameters remain unchanged. From these spectral curves shown in Supplementary Figure 12b, it is evident that the color collection efficiencies have similar profiles and the peak efficiencies of these three color lights are all larger than 50%, with different side lengths of nano-pillars. The experiment and simulation results have demonstrated that our metasurface color router is robust to fabrication errors.

To measure the intensity of three colors on the imaging plane, we illuminated the MBCR sample with broadband LED sources in the visible region, and use a custom microscope system to image the imaging plane of MBCR in Supplementary Figure 13. White light LED source (85-265VAC 50/60Hz, spectral information depicted in Supplementary Figure 14) illuminates the MBCR sample, with the imaging plane located at the working distance of a 100× objective (M Plan Apo, NA = 0.7, f=200). Another 10× objective (M Plan Apo, NA = 0.28, f=200) magnifies and projects the image onto a monochromic camera (ACA2500-14GC).

To calculate the color collection efficiency of the MBCR, light intensity distributions at different wavelengths shown in Supplementary Figure 15 are achieved. We first remove the background noise and define the color collection efficiency as the ratio of the intensity integral of transmittance in a

certain quarter period of the sample region to that in one period of the non-sample region, which is written as follows:

$$T_R = \frac{\int_{A_R} T_\lambda dxdy}{\int_{A_R+A_{G1}+A_{G2}+A_B} t_\lambda dxdy}, x, y \in [0, p/2],$$
$$T_{G1} = \frac{\int_{A_{G1}} T_\lambda dxdy}{\int_{A_R+A_{G1}+A_{G2}+A_B} t_\lambda dxdy}, x \in [0, p/2], y \in [p/2, p],$$
$$T_{G2} = \frac{\int_{A_{G2}} T_\lambda dxdy}{\int_{A_R+A_{G1}+A_{G2}+A_B} t_\lambda dxdy}, x \in [p/2, p], y \in [0, p/2],$$
$$T_B = \frac{\int_{A_B} T_\lambda dxdy}{\int_{A_R+A_{G1}+A_{G2}+A_B} t_\lambda dxdy}, x, y \in [p/2, p],$$

where T and t are transmittance of light incident from the sample region and transmittance of light incident from the non-sample region, respectively. λ is the working wavelength. p is the length of a unit cell consisting of an RGGB pattern. A_R, A_{G1}, A_{G2}, A_B is the area of responding color region in the imaging plane. Compared with the numerical simulations of MBCR, the actual measured peak efficiencies are 58%, 59% and 49% at the wavelengths of 640 nm, 540 nm, 460 nm, respectively, which is roughly consistent with that of simulation values (61%, 57%, 50%) with a=140 nm.

With the aiming to assess the performance of routing, we also calculate the tristimulus values (X, Y, Z) for a light source with spectral distribution T(λ), according to the equations as follows,

$$X = \int_{400}^{700} \bar{x}(\lambda) T(\lambda) d\lambda$$
$$Y = \int_{400}^{700} \bar{y}(\lambda) T(\lambda) d\lambda ,$$
$$Z = \int_{400}^{700} \bar{z}(\lambda) T(\lambda) d\lambda$$

where $\bar{x}(\lambda), \bar{y}(\lambda), \bar{z}(\lambda)$ are the CIE XYZ colour matching functions. X, Y and Z are normalized to obtain the chromaticity coordinates (x, y, z), as follows:

$$x = X/(X+Y+Z)$$
$$y = Y/(X+Y+Z) .$$
$$z = Z/(X+Y+Z)$$

Finally, the three chromaticity coordinates (x_R, y_R, z_R) and (x_G, y_G, z_G) and (x_B, y_B, z_B) in the 1931 CIE chromaticity diagram are calculated and shown in Supplementary Figure 17, using the spectral lines of three color pixels shown in Fig. 3f in the main text. In the diagram, colors obtained by MBCR (R1, G1, B1) and common CFs (R2, G2, B2) are represented in the color space, respectively. The color contrast of our RGB colors is lower than that of CFs, which can be attributed to the crosstalk between different colors. To solve this problem, we can insert a commercial Bayer CF below our MBCR to further eliminate this crosstalk. The schematic diagram is shown in Supplementary Figure 18a. By multiplying the measured spectra (see Fig. 3f in the main text) with the transmittance of commercial CFs (see Supplementary Figure 16), we can calculate the color collection efficiencies of this new design, which can both present the small color crosstalk of the CF and also keep the high efficiencies of MBCR, as shown in Supplementary Figure 18b. The chromaticity coordinates of this crosstalk cancellation scheme are marked with R3, G3, B3 in Supplementary Figure 17, with a better color contrast than that of the CFs. Since we only focus on a better single-layer CR with much higher efficiencies than the commercial BCF, such compound design will not be investigated in this work.

Supplementary Note 4. Imaging performance of MBCR

Furthermore, we also verify the imaging performance of the MBCR by setting up a simple imaging system, which is depicted in Supplementary Figure 19. A color picture is projected onto the collimation system through a small projector (XGIMI H3S). The collimating system includes two objective lenses and then scattered light from the projector becomes almost parallel light. An image was then created by the metasurface near its imaging plane through focusing light coming from the projector. A microscope consisting of two objective lenses and a monochromic camera (ACA2500-14GC) captures this image and can adjust the magnification of the image. Before image capture, the camera’s dark noise was subtracted after taking a sequence of pictures with the lens cap on. However, to obtain imaging results of BCF, we insert R, G, and B CFs (Thorlabs FD1R, FD1G and FD1B, see Supplementary Figure 16) alternately in the imaging setup, and measure the corresponding monochromic image of each filter. Then, we virtually constructed R, G, and B pixels according to the three measured images and the arrangement of the RGGB pattern to simulate the BCF spectrum. Finally, a color image coming from BCF is created according to the constructed

pixels.

Supplementary Figure 20a and Supplementary Figure 20b are the raw image captured by the monochromic imaging sensor with different resolutions in order to show different levels of routing details. During the reconstruction procedure of color imaging, due to the inhomogeneity of each pixel, combining four pixels of RGGB into one pixel directly would introduce the noise of the background light. Thus, we first average the intensity of each pixel for the captured monochromic image, and then convert a set of RGGB mosaic patterns into a greyscale image shown in Fig. 4b of the main text. The spectral responses of the MBCR are then imposed on each pixel using the method of conversion matrix. The conversion matrix directly converts the detected intensity of spectral values into three-channel RGB values. In brief, the desired RGB values can be modeled as $Y = AX$, where Y is the RGB signal vector and X is the detected photocurrent vector, and A is the conversion matrix (3×3). In order to solve the conversion matrix A, we need to first calibrate the relationship between the photocurrent vector X and the associated RGB signal vector Y. Then the conversion matrix A can always be used to recalculate the three-channel RGB values Y from the detected spectral values X of any unknown target image. Thus, a color figure with the RGGB mosaic pattern is obtained as seen in Supplementary Figure 20c and Supplementary Figure 20d. Finally, the demosaicing process is implemented to obtain the three-channel color image. The enhancement factor of brightness between MBCR and CF is defined as the ratio of the average brightness of the two whole greyscale images, and it is calculated to be 2.39. Supplementary Figure 21 shows the ratio of the brightness of the greyscale image for these two figures, and the ratio shows some slight fluctuations due to the brightness saturation of the captured figure from MBCR, leading to the slightly low ratio at the blank area of the figure.

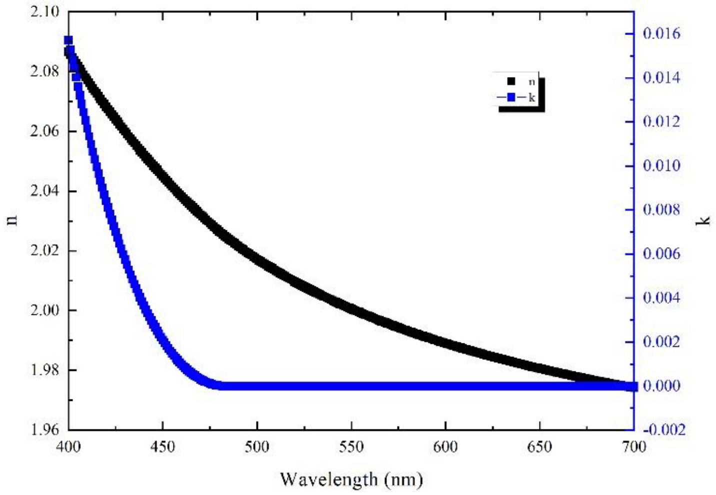

Supplementary Figure 1. The refractive index of Si_3N_4 in the visible regime.

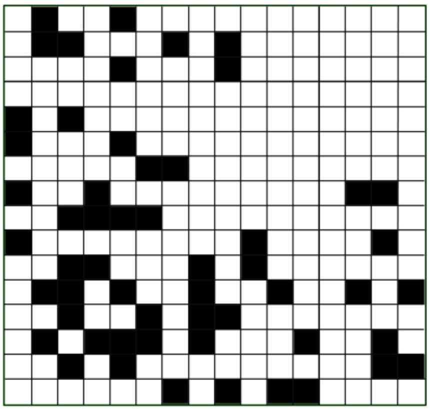

Supplementary Figure 2. Binary distribution map of the optimized metasurface in the main text with a thickness of 600 nm. The black squares represent Si_3N_4 and the whites represent air.

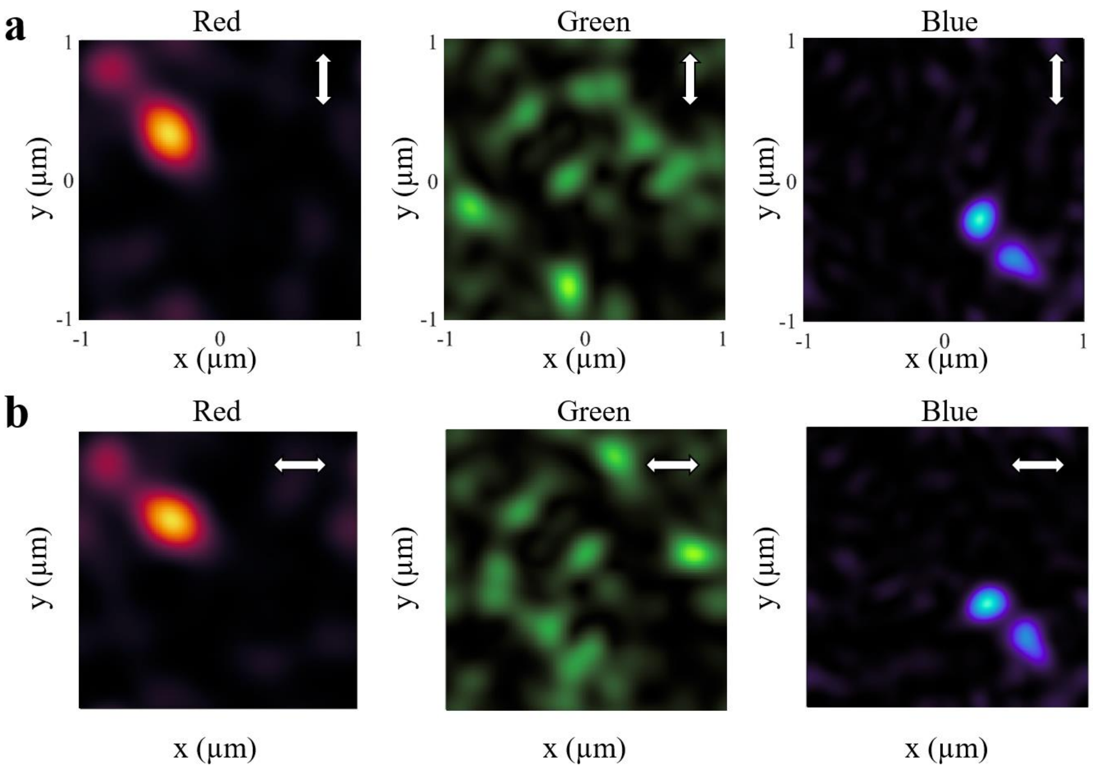

Supplementary Figure 3. Power flow density distribution maps of the transmitted light on the focal plane for vertical polarization (a) and horizontal polarization (b) at 660 nm, 540 nm and 450 nm, respectively.

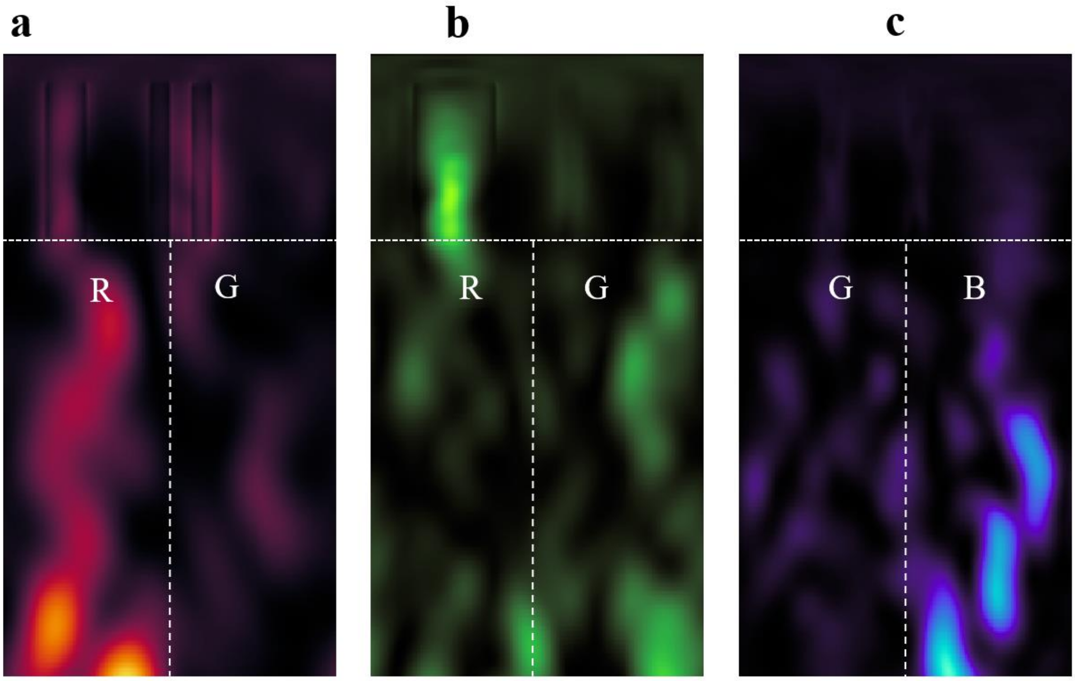

Supplementary Figure 4. Cross-sectional view (XZ plane) of the pointing vector distributions $|P|$ at

λ =660 nm (a), 540 nm (b), and 450 nm (c) at the locations of y=-0.6 μm, 0.2 μm, 0.5 μm. The white dash lines represent the interface between Si_3N_4 metasurface and quartz substrate or the interface of different color pixels in the Z direction. Letters R, G, B represent the specific color pixel.

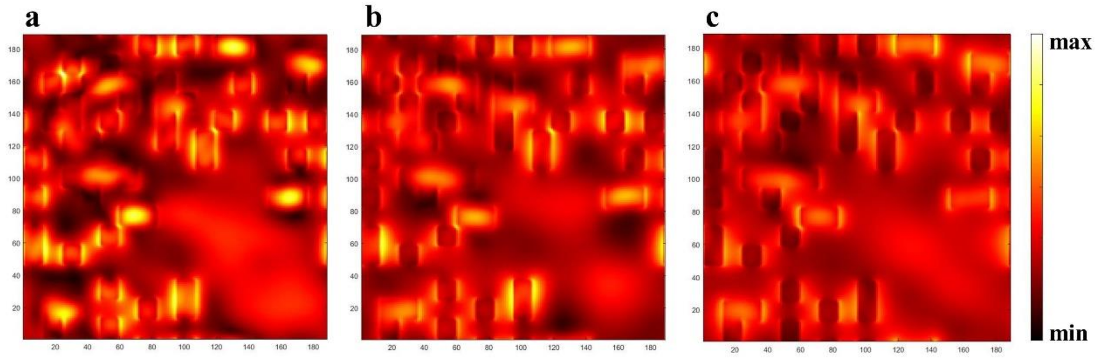

Supplementary Figure 5. Top view of the electric field $|E|$ distributions in the metasurface at λ =450 (a) nm, 540 nm (b), and 660 nm (c).

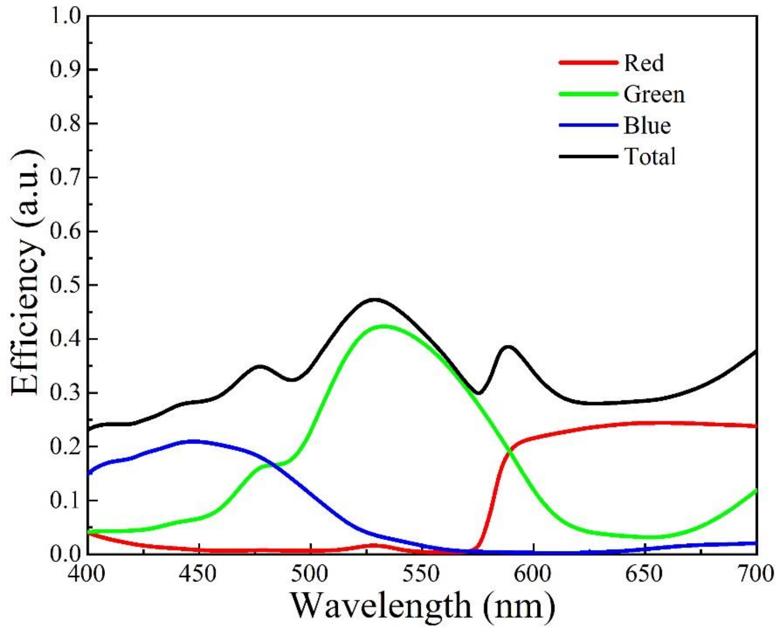

Supplementary Figure 6. The color collection efficiencies calculated by the transmittance curves of commercial Bayer CFs (*https://www.fujifilm.com/us/en/business/semiconductor-materials/image-sensor-color-mosaic/rgb/applications*).

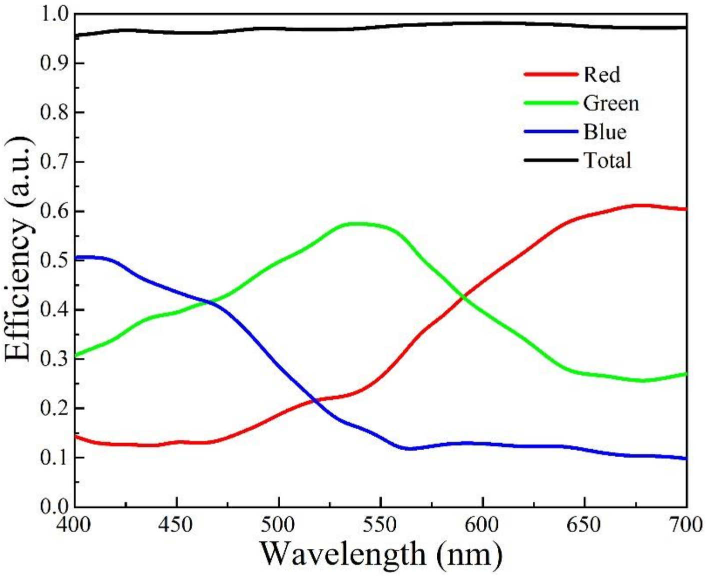

Supplementary Figure 7. The total energy efficiency calculated theoretically of the MBCR, respectively.

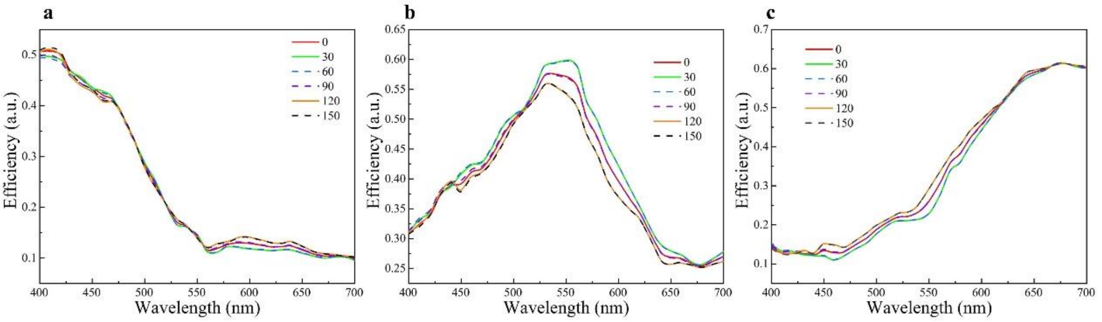

Supplementary Figure 8. Transmittance curves of our MBCR for blue (a), green (b) and red (c) pixels when considering the angle of polarization (0-180°).

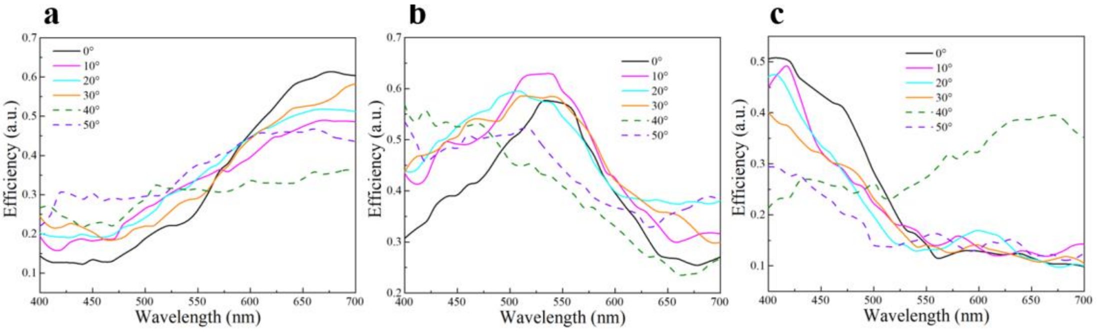

Supplementary Figure 9. Transmittance curves of our MBCR for red (a), green (b) and blue (c) pixels when considering the angle of incidence up to 50°.

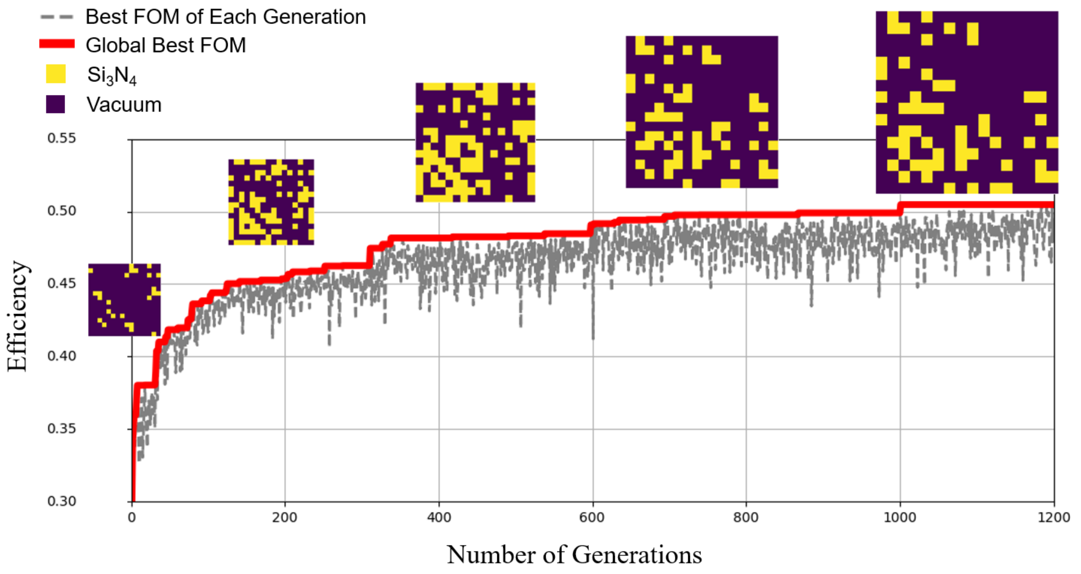

Supplementary Figure 10. Figure of merit (FOM) as a function of iterations during the process of optimization. Insets: the pattern examples generated during optimization.

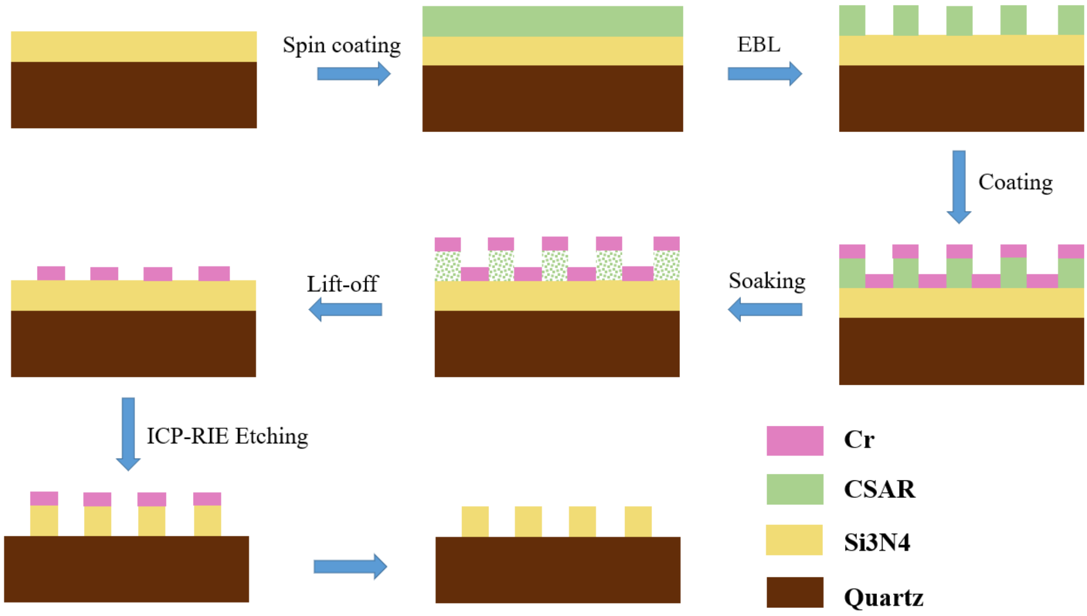

Supplementary Figure 11. Flow chart of the preparation of the MBCR sample.

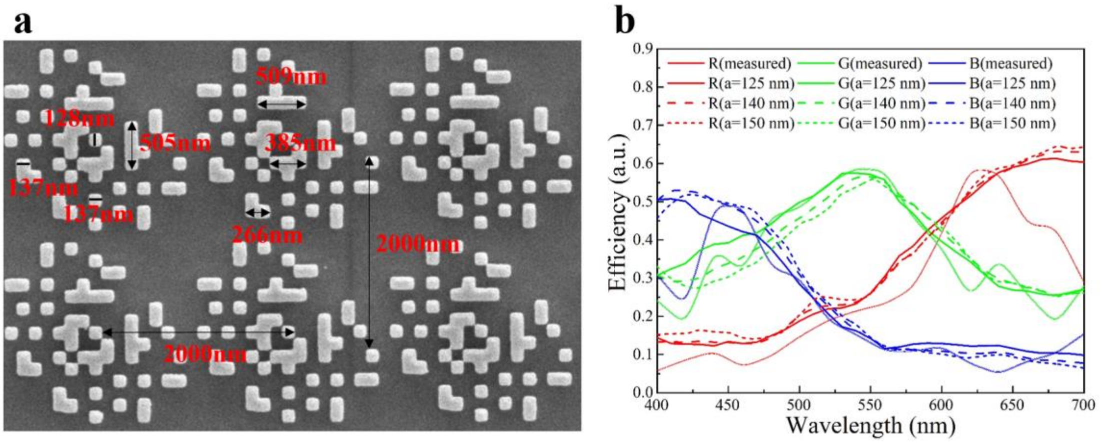

Supplementary Figure 12. (a) Measurement of the fabricated sample by proportional scaling. (b) Comparison between experiment and simulation (with variable side lengths of the pillars) results of RGB collection efficiencies in the visible region.

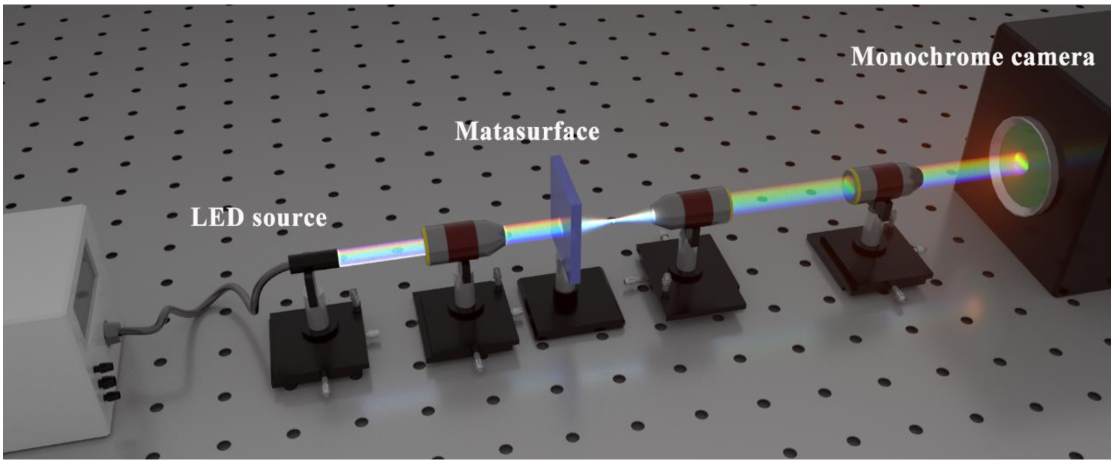

Supplementary Figure 13. Experimental setup for characterizing the focal plane and light splitting effect of our MBCR.

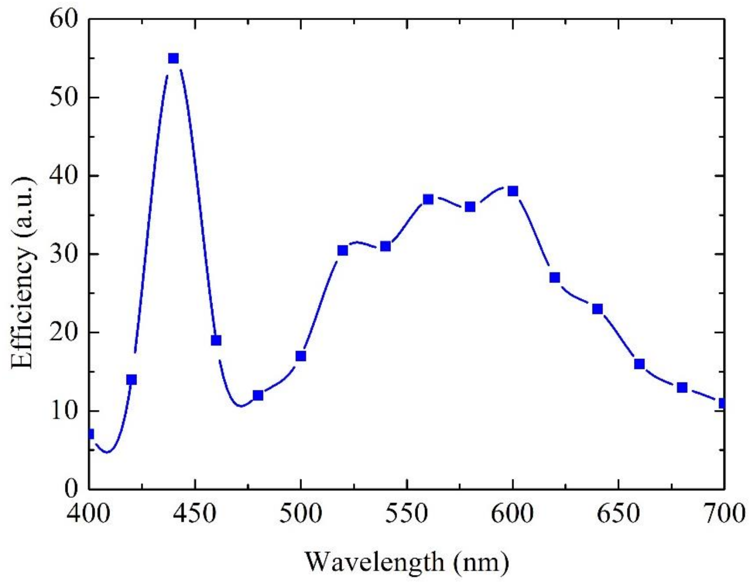

Supplementary Figure 14. Spectral information of LED light used in the experiment setup for characterizing the light splitting effect.

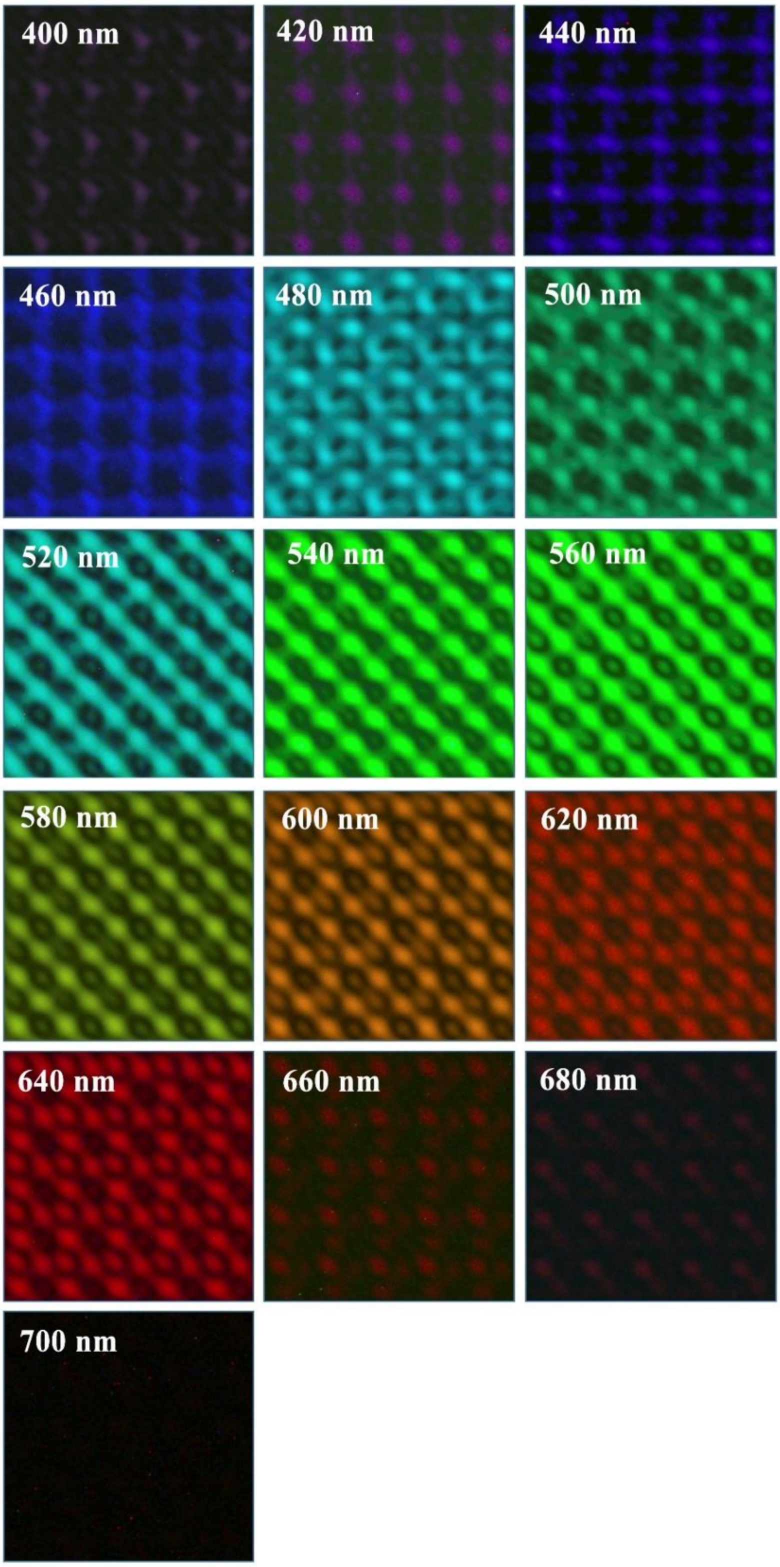

Supplementary Figure 15. Measured light intensity distribution on the imaging plane under different wavelengths of incident light on a linear scale with the MBCR. The wavelength of the incident light is labeled above each picture.

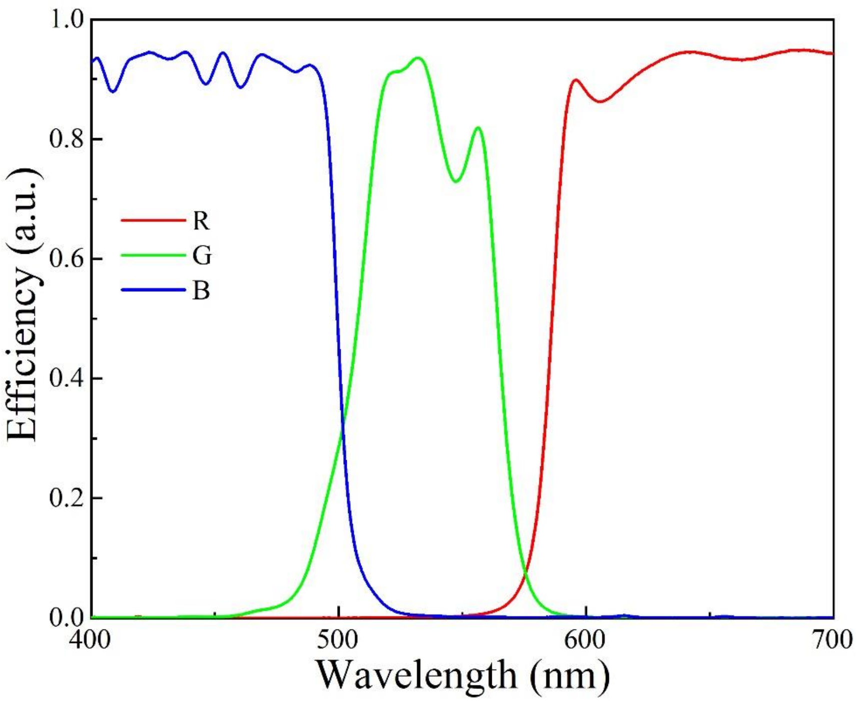

Supplementary Figure 16. Transmittance curves of common CFs (Thorlabs FD1R, FD1G and FD1B).

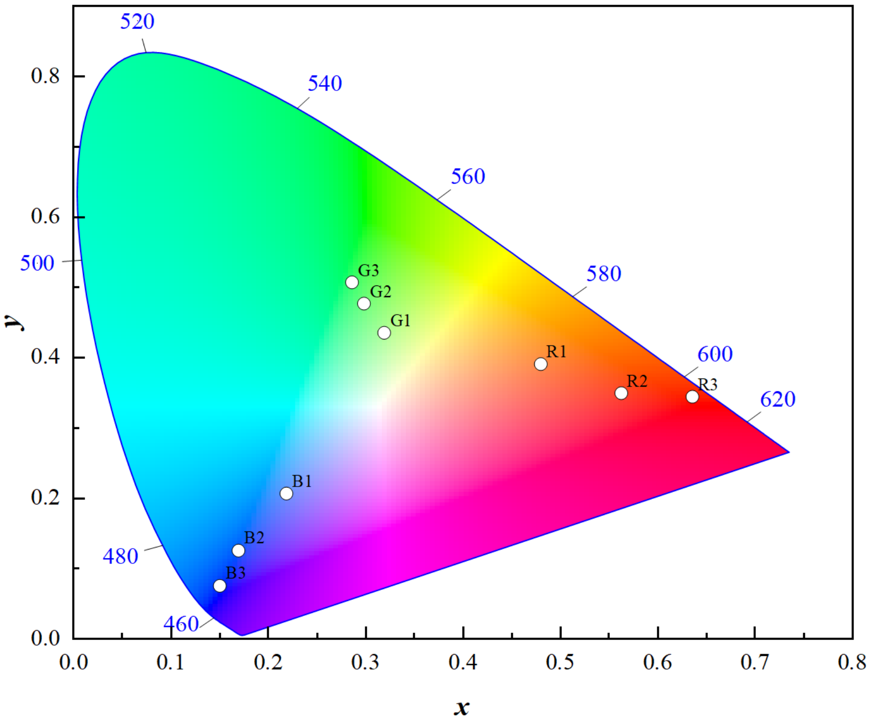

Supplementary Figure 17. Characterization of colors obtained by MBCR (R1, G1, B1), commercial CFs (R2, G2, B2) and the crosstalk cancellation scheme that places metasurface on top of BCF (R3, G3, B3) in the 1931 CIE chromaticity diagram.

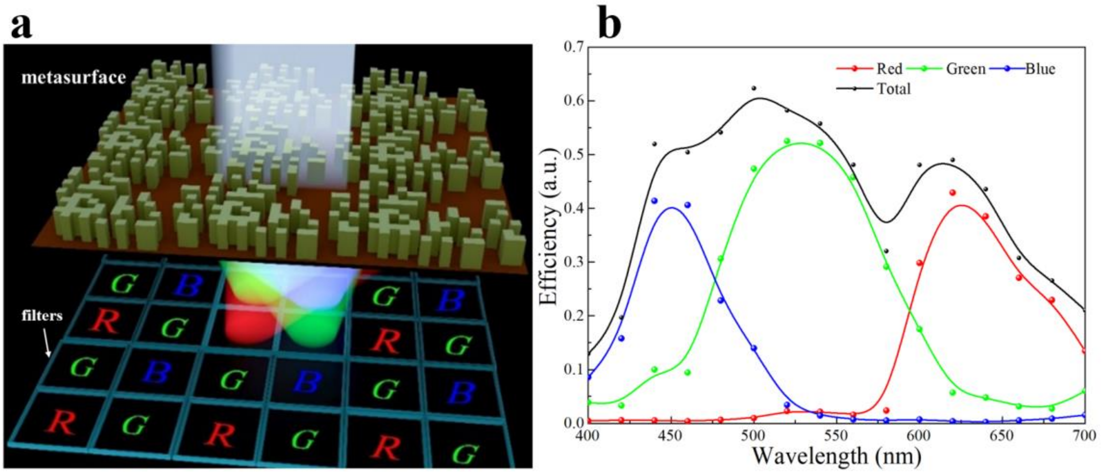

Supplementary Figure 18. (a) Schematic of the crosstalk cancellation scheme that combines MBCR and CFs together. (b) The measured color collection efficiencies of each channel in the visible region for the crosstalk cancellation scheme. Each color curve corresponds to its color channel. The black line represents the energy utilization efficiency.

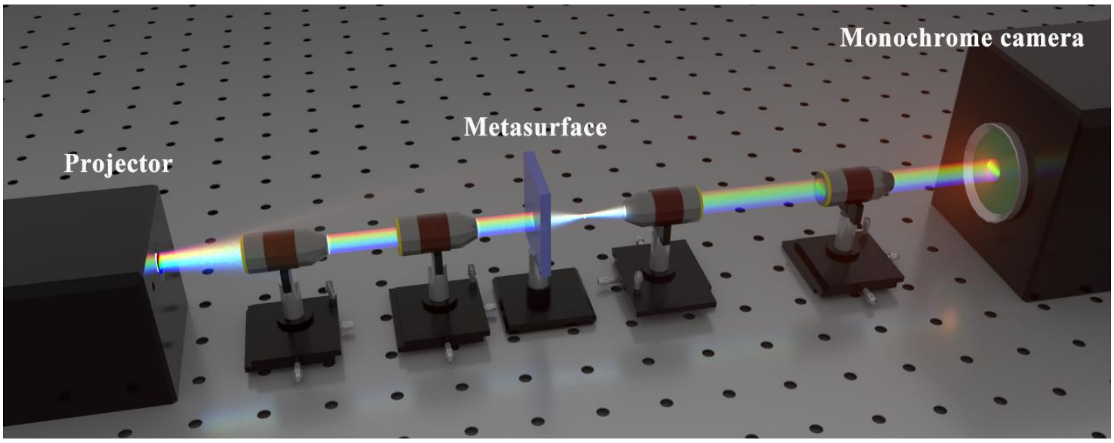

Supplementary Figure 19. Experimental setup for imaging with MBCR.

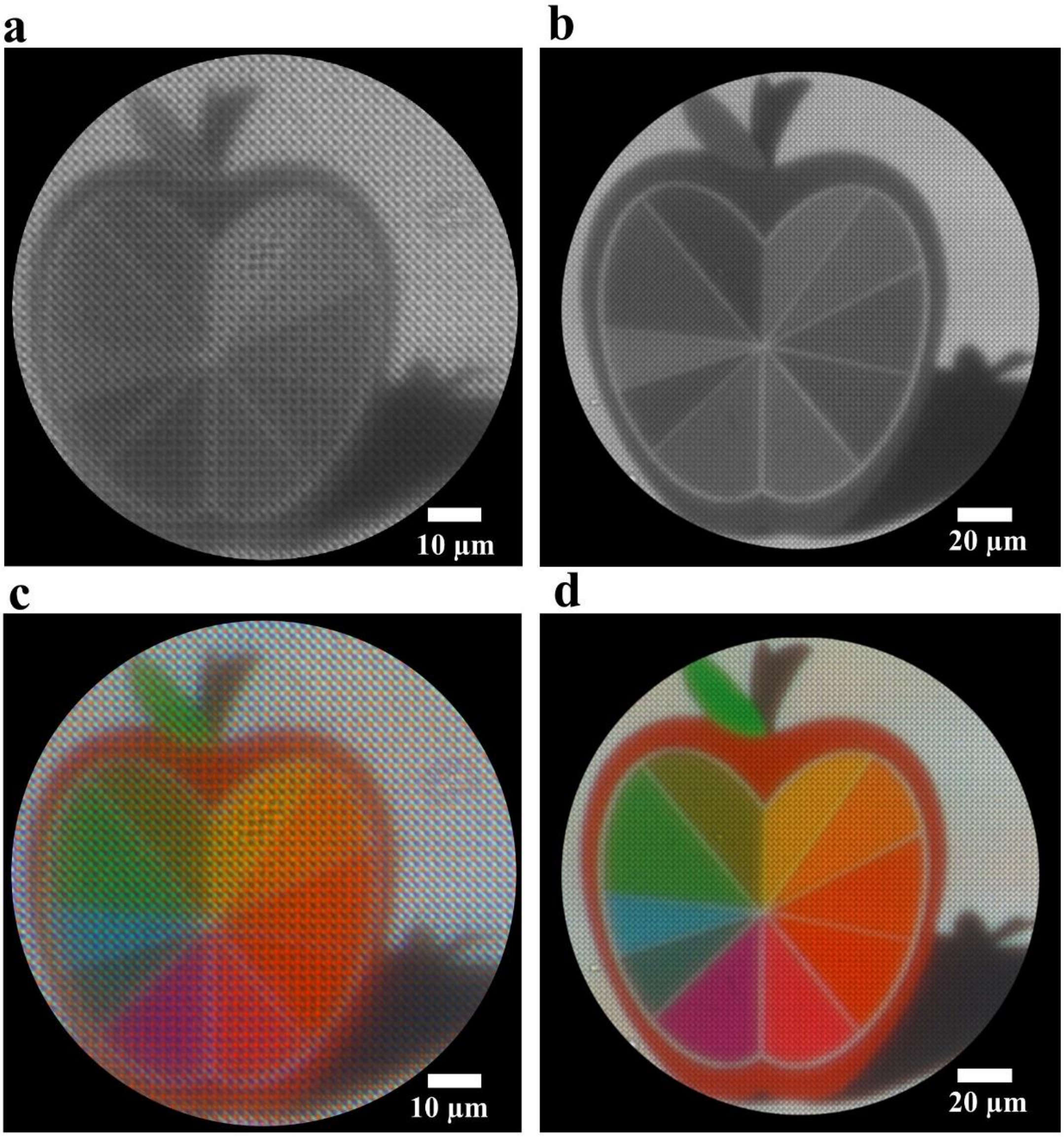

Supplementary Figure 20. (a)(b) Monochrome: The imaging result of the target objects obtained by the greyscale camera under white LED light with different resolutions. (c)(d) Color: Display of the target objects produced by adding spectral information of LED light with different resolutions.

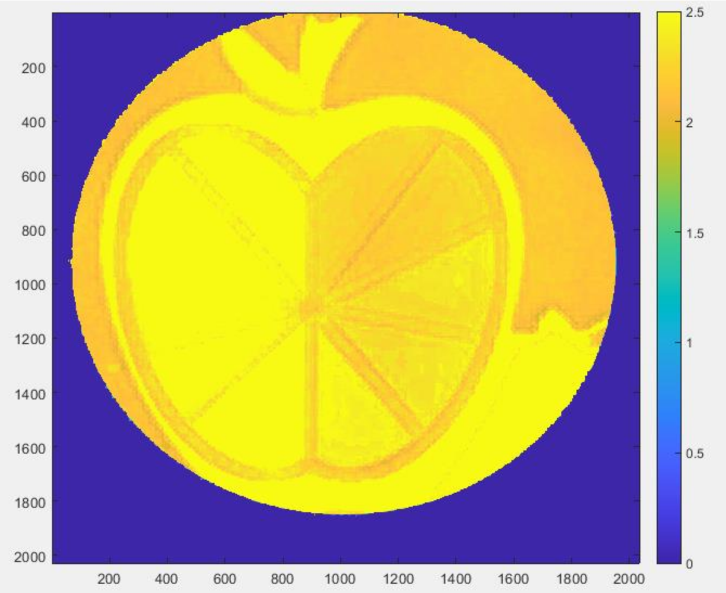

Supplementary Figure 21. The calculated enhancement of brightness for the MBCR compared to CF.

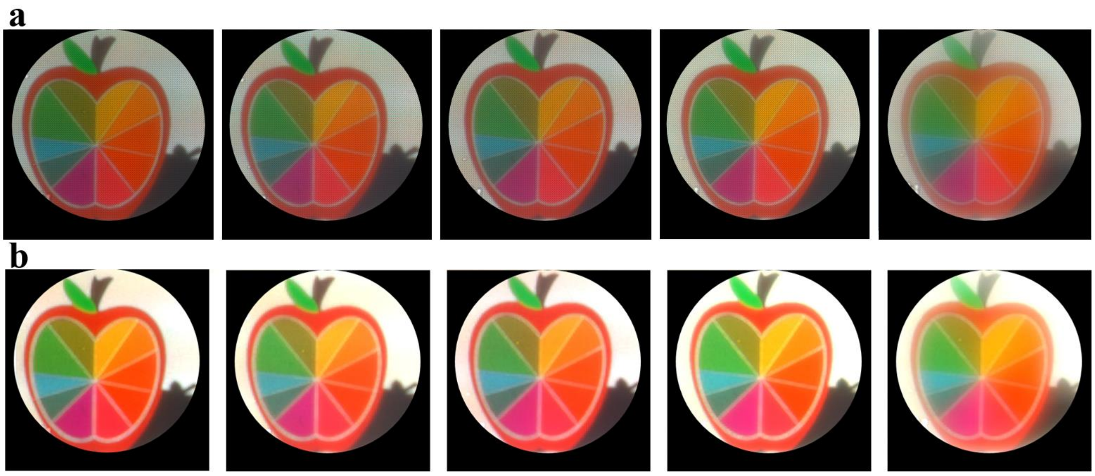

Supplementary Figure 22. Influence of numerical aperture (NA) on imaging performance. (a) Images taken at different numerical aperture (NA) of 0.02, 0.05, 0.09, 0.14, 0.19. (b) Image reconstruction of the pictures in (a).
